# Joint Genetic Analyses of Mitochondrial and Y-Chromosome Molecular Markers for a Population from Northwest China

**DOI:** 10.3390/genes11050564

**Published:** 2020-05-18

**Authors:** Yuxin Guo, Zhiyu Xia, Wei Cui, Chong Chen, Xiaoye Jin, Bofeng Zhu

**Affiliations:** 1 Key Laboratory of Shaanxi Province for Craniofacial Precision Medicine Research, College of Stomatology, Xi’an Jiaotong University, Xi’an 710004, China; guoyuxin_004@stu.xjtu.edu.cn (Y.G.); cuiwei3702@stu.xjtu.edu.cn (W.C.); cc18883368974@stu.xjtu.edu.cn (C.C.); jinxy0901@stu.xjtu.edu.cn (X.J.); 2 Clinical Research Center of Shaanxi Province for Dental and Maxillofacial Diseases, College of Stomatology, Xi’an Jiaotong University, Xi’an 710004, China; 3 College of Medicine & Forensics, Xi’an Jiaotong University Health Science Center, Xi’an 710061, China; 4 Department of Epidemiology, University of Washington, Seattle, WA 98105, USA; zhiyuxia@uw.edu; 5 Multi-Omics Innovative Research Center of Forensic Identification, Department of Forensic Genetics, School of Forensic Medicine, Southern Medical University, Guangzhou 510515, China

**Keywords:** mtDNA, Y-STRs, independence test

## Abstract

The genetic markers on mitochondria DNA (mtDNA) and Y-chromosome can be applied as a powerful tool in population genetics. We present a study to reveal the genetic background of Kyrgyz group, a Chinese ethnic group living in northwest China, and genetic polymorphisms of 60 loci on maternal inherited mtDNA and 24 loci on paternal inherited Y-chromosome short tandem repeats (Y-STRs) were investigated. The relationship between the two systems was tested, and the result indicated that they were statistically independent from each other. The genetic distances between Kyrgyz group and 11 reference populations for mtDNA, and 13 reference populations for Y-STRs were also calculated, respectively. The present results demonstrated that the Kyrgyz group was genetically closer to East Asian populations than European populations based on the mtDNA loci but the other way around for the Y-STRs. The genetic analyses could largely strengthen the understanding for the genetic background of the Kyrgyz group.

## 1. Introduction

As one of the populous countries in the world, China consists of 56 official ethnic groups, some of them, including the Uygur, Kazak, Uzbek, and Kyrgyz groups, live in northwest China [1]. On the basis of molecular genetic markers on human autosomes [2,3,4,5,6], the previous studies indicated that these ethnic groups had ancestral components of East Asian and European populations. There are more than 186,000 Kyrgyz individuals (https://guides.lib.unc.edu/china_ethnic/statistics) in China. Based on two types of autosomal markers, insertion/deletion (InDel) [3] and short tandem repeat (STR) loci [4], our previous studies revealed that the Kyrgyz group was genetically closer to other ethnic groups in northwest China. The current research was conducted on the Kyrgyz group using genetic markers of maternal inherited mitochondria DNA (mtDNA) and paternal inherited Y-chromosome STRs (Y-STRs) in order to reveal the genetic relationships between Kyrgyz group and different reference populations from the maternal and paternal perspective [7,8]. 

The marker system on mtDNA can be applied as a useful tool for individual identification, maternal lineage test, and ancestry inference in forensic applications [9,10,11]. Its rapid mutation rate, lacking recombination, maternal inheritance and high polymorphism, make mtDNA loci the reasonable marker system for analyzing maternal genetic relationships among different ethnic groups. Compared with nuclear DNA, mtDNA has great advantages in forensic applications for testing highly degraded, damaged, or trace amounts of biological samples [12] due to its high copy numbers per cell, which improves the success rate of mtDNA detection [11].

Another marker system used in this study is Y-STRs, which contribute to plenty of Y chromosomal haplotypes for forensic applications, historical researches, and genealogical investigation of paternal relatives [13,14]. Based on that, there is a large public database, named the Y-STR Haplotype Reference Database (YHRD, https://yhrd.org), which contains a large number of Y-STR haplotypes from different populations all over the world, can be used as reference population data.

The purpose of the current study was to further understand the historical background of the Kyrgyz group and its paternal and maternal genetic relationships with various populations. We also conducted an independence test between the two marker systems in this study based on the method, an extension of contingency chi-square tests for linkage disequilibrium [15], as suggested by Dr. Bruce Weir from the University of Washington.

## 2. Methods and Materials

### 2.1. Sample Collections

To detect the mtDNA loci and Y-STRs, we collected the blood samples of unrelated healthy individuals from Kyrgyz group in the Kizilsu Kirghiz Autonomous Prefecture, in the northwest of the People’s Republic of China. *N*_m_ (*N*_m_ = 138) samples were detected by the mtDNA marker system, *N*_y_ (*N*_y_ = 241) male samples were detected by the Y-STR marker system, and *N*_j_ (*N*_j_ = 88) samples were detected by both of the two marker systems. This study was performed in accordance with the humane and ethical research principles approved by the ethical committee of Xi’an Jiaotong University Health Science Center, China. All the reference population data for mtDNA and Y-STRs were collected from previously published researches.

### 2.2. Amplification and Genotyping

We detected 60 mtDNA loci using a commercial kit named Expressmarker mtDNA-SNP 60 (AGCU ScienTech Incorporation, Jiangsu, China), which contained 58 mtDNA single nucleotide polymorphisms (SNPs) including nt152, nt709, nt1541, nt1719, nt1811, nt2706, nt3010, nt3348, nt3970, nt4216, nt4491, nt4833, nt4883, nt5178, nt5417, nt5442, nt5460, nt6446, nt7028, nt7196, nt7600, nt8020, nt8414, nt8584, nt8684, nt8697, nt8701, nt8793, nt8794, nt8964, nt9123, nt9477, nt9545, nt9698, nt9824, nt10310, nt10397, nt10398, nt10400, nt10873, nt11215, nt11251, nt11719, nt12372, nt12705, nt12811, nt13104, nt13928, nt14569, nt14668, nt15043, nt15784, nt16126, nt16129, nt16311, nt16316, nt16319, nt16362), an InDel locus (DEL, the 9bp deletion of CCCCCTCTA sequence; NORM, non-deletion of 9bp), and a (CA)n locus (nt514-nt524). These 60 loci were separately amplified with three multiplex amplification panels in three independent 10ul volume systems according to the manufacturer’s instruction [16], analyzed using capillary electrophoresis platform performed by ABI Prism 3500xL Genetic Analyzer (Applied Biosystems, Foster City, CA, USA), and genotyped by GeneMapper^®^ ID-X software v 1.3 (Applied Biosystems, Forster City, CA, USA).

Another commercial kit, Microreader^™^ 24Y Direct ID System (Microread Genetics Ltd., Jiangsu, China) was applied to detect 24 Y-STRs, containing 23 overlapped loci with PowerPlex Y23 System (DYS393, DYS570, DYS19, DYS392, DYS549, YGATAH4, DYS458, DYS481, DYS635, DYS448, DYS533, DYS456, DYS389I, DYS390, DYS389II, DYS438, DYS576, DYS391, DYS439, DYS437, DYS385a/b, and DYS643) and DYS460 loci. Multiplex amplification was conducted in a 10ul volume system as descried in the manufacturer’s instruction [17]. Twenty-four Y-STRs were analyzed and genotyped by the same platform and software as the detection of 60 mtDNA loci.

In this research, M4615 (Microread Genetics Ltd., Jiangsu, China) and 9947A (Promega, Madison, WI, USA) were used as control DNA in the genotyping of Y-STRs and mtDNA loci, respectively. And ddH_2_O was used as negative control.

### 2.3. Statistical Analyses

For mtDNA loci, the two forensic parameters, haplotype diversity (HD_m_) and nucleotide diversity (ND_m_), are calculated by DnaSP version 5.0 [18], respectively. Random matching probability (RMP_m_) and discrimination power (DP_m_) are calculated by the formula (1) below [19].

(1) $${\mathrm{RMP}}_{m}=\sum P_{mhi}^{2}$$

where $P_{mhi}$ indicates haplotype frequency of the i-th haplotype on mtDNA in a certain population.

Gene diversity (GD_y_), haplotype diversity (HD_y_), and matching probability (MP_y_) for Y-STRs are measured using the following formulas (2), (3) and (4) [19]:

(2) $$\mathrm{GD}y_{\;}=\frac{N_{y}}{N_{y}-1}\left(1-\sum P_{yai}^{2}\right)$$

(3) $$\mathrm{HD}y_{\;}=\frac{N_{y}}{N_{y}-1}\left(1-\sum P_{yhi}^{2}\right)$$

(4) $$\mathrm{MP}y_{\;}=\sum P_{yai}^{2}$$

where $N_{y}$ indicates the total number of individuals who are detected by the Y-STR marker system in a population, $P_{yai}$ is the allele frequency of the i-th allele of locus “a” on Y chromosome, and $P_{yhi}$ is the haplotype frequency of the i-th haplotype on Y chromosome in a population.

After aligning with the revised Cambridge Reference Sequence (rCRS) [20], the genotyping results of mtDNA loci except (CA)n were classified into various haplogroups [8]. Meanwhile, the genetic distance, pairwise *Fst* values, for 58 mtDNA SNPs except 9bp and (CA)n among Kyrgyz group and other reference populations were estimated by Arlequin version 3.0 [21]. For Y-STRs, the Y-STR haplogroups were predicted by NevGen Genealogy Tools version 1.1 (https://www.nevgen.org/). In addition, another value of genetic distance for 23 Y-STRs except DYS460, i.e. pairwise *Rst* values [22], were estimated online (https://yhrd.org/amova) based on YHRD database. Plots of neighbor-joining (NJ) tree and multidimensional scaling analysis (MDS) were drawn by MEGA version 6.06 (http://megasoftware.net/) and PASW STATISTICS version 18.0 (https://www.ibm.com/products/spss-statistics) based on pairwise *Fst* values for 58 mtDNA SNPs and pairwise *Rst* values for 23 Y-STRs. 

The test of independence between the two systems was conducted by *R* statistical software version 3.0.2 (https://www.r-project.org/). The 88 individuals detected by both systems were studied in this part. For each pair of individuals, the number of Y-STRs with the same genotypes was recorded as y, ranged from 0 to 24, and the number of mtDNA loci except 9bp and (CA)n with the same genotypes was recorded as m, which ranged from 0 to 58. There were 3828 distinct pairs of 88 individuals in total, therefore, we had the values of $y_{i}$ and $m_{i}$, where i=1, 2, …3828. A Fisher’s exact test [23] by *R* function (fisher.test {stats}) for independence test between Y-STRs and mtDNA SNPs was performed based on the matrix formed by $y_{i}$ and $m_{i}$.

## 3. Results

### 3.1. Forensic Parameters

Allele frequencies of 60 mtDNA loci in 138 Kyrgyz individuals are shown in Table S1. According to the results, most of the mtDNA SNPs (48 out of 58 SNPs) were detected as transition, two SNPs (nt5178 and nt7196) were identified as transversion, and eight SNPs (nt1541, nt3348, nt3970, nt6446, nt8697, nt8793, nt8964, and nt13928) were found to be no polymorphisms. In Table S2, the 60 mtDNA loci defined 55 haplotypes and 28 haplogroups except (CA)n for haplogroup analysis. The RMP_m_ and DP_m_ values calculated on the basis of the number of haplotypes were 0.0807 and 0.9193. In addition, the values of HD_m_ and ND_m_ were 0.9838 +/− 0.0035 and 0.1820 +/− 0.0929.

Allele frequencies and GD_y_ and MP_y_ values for 24 Y-STRs in 241 individuals are listed in Table S3. In total, 184 alleles on 24 Y-STRs were observed with their allele frequencies ranging from 0.0041 to 0.8631. The GD_y_ values on 24 Y-STRs ranged from 0.2468 (DYS393) to 0.7653 (DYS389 II). A total of 152 haplotypes and 27 haplogroups were defined by the 24 Y-STRs, which contained 23 Y-STRs overlapped with PowerPlex Y23 System (Promega, Madison, USA), respectively (Table S4). Calculated from the haplotype frequencies, the HD_y_ value was equal to 0.9863.

### 3.2. Haplogroup Analyses for 59 mtDNA loci

**Figure 1:**
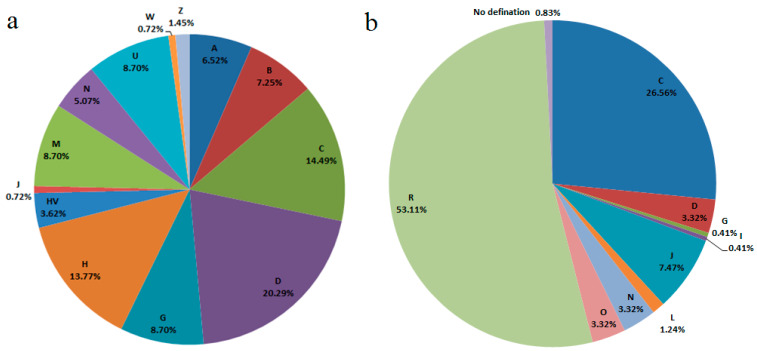
The pie charts for the haplogroup frequencies of the Kyrgyz group, (**a**) based on 59 mtDNA loci in 138 individuals, haplogroup M in this plot excluded haplogroups C, D, G and Z; haplogroup N in this plot excluded haplogroups A, B, J, H, HV, U and W; (**b**) based on 23 Y-STRs in 241 male individuals.

The haplogroup results for 59 mtDNA loci in 138 individuals were listed in Table S2. A pie chart of mtDNA haplogroup frequencies was shown in Figure 1a. The highest frequencies of mtDNA haplogroups were haplogroup C (14.49%) and haplogroup D4 (14.49%), followed by haplogroup H (13.77%) in Kyrgyz individuals. The largest haplogroup was observed as haplogroup D lineages, which included haplogroups D4 (14.49%), D4b (1.45%), D4e (1.45%), and D5 (2.90%), accounting for 20.29% in total.

According to the haplogroup geographic affiliations by Underhill and Kivisild [24], most of the mtDNA haplogroups in the 138 Kyrgyz individuals, which were more common in East Asian populations, were haplogroup D (20.29%) including haplogroups D4, D4e, D4b, and D5; haplogroup C (14.49%); haplogroup G (8.70%); haplogroup M (8.70%, except haplogroups C, D, G and Z) including haplogroups M*, M7b1a1, M7c2, M8a, M9, and M9a; haplogroup B (8.70%) including haplogroups B4’5, B4a, and B5; haplogroup A (6.52%); haplogroup N (2.17%, except haplogroups A, B, J, H, HV, U and W) including haplogroups N9 and N9a; and haplogroup Z (1.45%). The remaining haplogroups in the studied Kyrgyz group accounted for 30.43%, which were widely dispersed in European and South Asian populations in different ratios, and included haplogroup H (13.77%); haplogroup U (8.70%) including haplogroups U1, U2’3’4’7’, U5a’b, and U7; haplogroup HV (3.62%); haplogroup N (2.90%) including haplogroup N1a1; haplogroup J (0.72%); and haplogroup W (0.72%). A majority of the mtDNA haplogroups in the studied group was common in East and South Asian populations. These haplogroups, which were often observed in East Asian populations, accounted for over two thirds (69.57%) in the studied group, which implied critical maternal influence from East Asian populations.

### 3.3. Haplogroup Analyses for 23 Y-STRs

The Y-STR haplogroup distributions in 241 male individuals were listed in Table S4 and shown in Figure 1b. The frequency of the R1a haplogroup was the highest (48.13%), followed by the C2 M217 >> F1918 haplogroup (24.07%). The haplogroup R lineages had the dominant frequency with a total of 53.11%, consisting of haplogroups R1a (48.13%), R1b (0.83%), R1b M73 (3.32%), and R2 M479 (0.83%). The total frequency of haplogroup C lineages took the second place (26.56%), which included haplogroups C2 M217 misc (0.83%), C2 M217 > F1067 (0.83%), C2 M217 >> F1918 (24.07%), and C2b1a1b1 F3985 (0.83%), respectively.

The geographic affiliations of the Y-STR haplogroups for the paternal lineages in this study were different from those of the mtDNA haplogroups for the maternal lineages. The Y-STR haplogroup with the highest frequency in Kyrgyz individuals was haplogroup R1a (48.13%), which was mostly prevalent in European and South Asian populations, according to previously reported researches [25,26,27]. Haplogroup C-M217, mostly distributed in East Asian and American populations, also occurred frequently in the Kyrgyz group (with the frequency of 25.73%). The haplogroups J2, R1b, J1, G*, and I1 which were more common in European populations were 6.64%, 4.15%, 0.83%, 0.41%, and 0.41% in the studied Kyrgyz group, respectively [28], and the haplogroups J2 and J1 also occurred in South Asian and Sub-Saharan African populations, respectively [24]. Nevertheless, the Y-STR haplogroups that were mainly prevalent in East Asian populations presented at lower frequencies in Kyrgyz individuals, including the haplogroups D1 (3.32%, also in North Asian populations), N1 (3.32%), O2a (2.90%, also in South Asian populations) and O1 (0.41%). Compared with the maternal inheritance of mtDNA, the males of the Kyrgyz group kept more paternal ancestry component from European populations.

### 3.4. Population Genetic Differentiation Analyses for mtDNA

**Figure 2:**
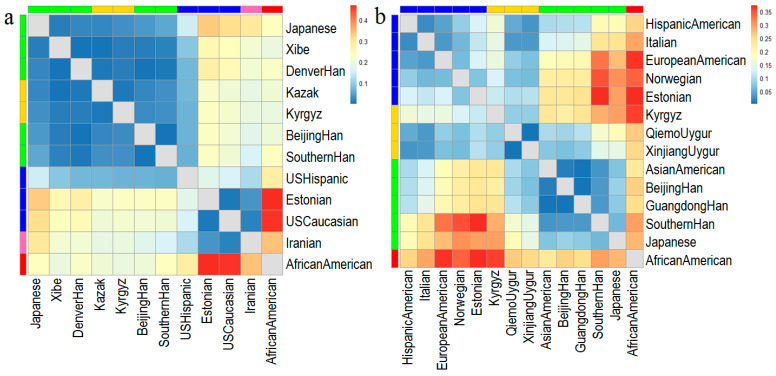
The two heatmaps for genetic distances among the Kyrgyz group and the reference populations, (**a**) pairwise *Fst* values based on 58 mtDNA SNPs among 12 populations; (**b**) pairwise *Rst* values based on 23 Y-STRs among 14 populations.

**Figure 3:**
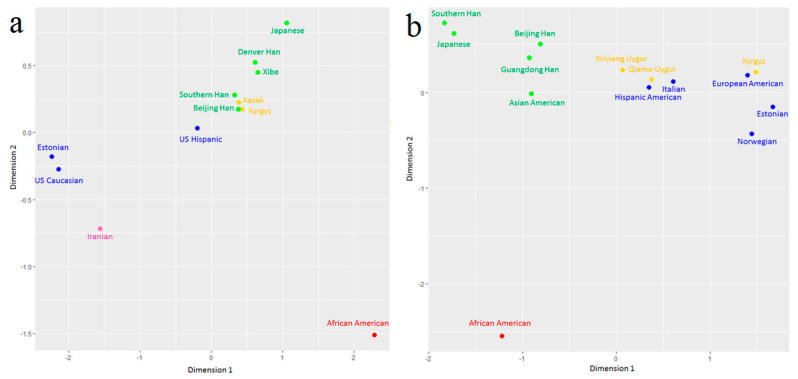
The multidimensional scaling analysis (MDS) plots for Kyrgyz group and reference populations, (**a**) based on the same of 58 mtDNA SNPs; (**b**) based on the same of 23 Y-STRs.

**Figure 4:**
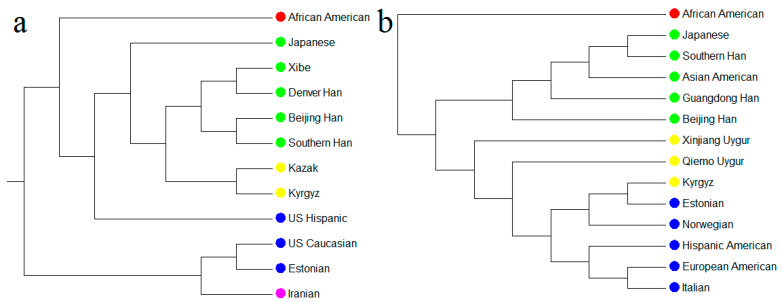
The neighbor-joining (NJ) trees for the Kyrgyz group and reference populations, (**a**) based on the same of 58 mtDNA SNPs; (**b**) based on the same of 23 Y-STRs.

The pairwise *Fst* values for 58 mtDNA SNPs were calculated between the Kyrgyz group and 11 reference populations (East Asian populations: Beijing Han, Southern Han, Denver Han, Japanese [29], Xibe [30], and Kazak in northwest China [16]; European populations: Estonian [11], Hispanic, and Caucasian [31]; West Asian population: Iranian [32]; and African population: African American [31]). The pairwise *Fst* values were graphically represented by a heatmap in Figure 2a, which were indicated by gradient colors from blue (low values) to red (high values), ranging from 0.0007 (between Beijing Han and Sothern Han populations) to 0.4677 (between African American and Estonian populations). We also plotted the MDS (Figure 3a) and NJ tree (Figure 4a) based on the pairwise *Fst* values. In Figure 3a, the Kyrgyz and Kazak ethnic groups, located at the central area, were genetically close to each other and surrounded by other East Asian populations. There were three main branches on the NJ tree in the Figure 4a, in which the top branch had only one population (African American); the middle branch included Kazak, Kyrgyz, other East Asian populations, and a European population; and the bottom branch consisted of European and West Asian populations. In the middle branch, the Kyrgyz group was first clustered with Kazak ethnic group, and then with Xibe, Southern Han, Beijing Han, and Denver Han populations, and finally with Japanese and Hispanic populations.

### 3.5. Population Genetic Differentiation Analyses for Y-STRs

As shown in Figure 2b, a heatmap of the pairwise *Rst* values for 23 Y-STRs indicated the genetic relationships of Kyrgyz group and 13 reference populations. These 13 reference populations included East Asian populations: Beijing Han [33,34,35], Guangdong Han [36], Southern Han [37], Asian American [38], Japanese [39,40,41,42], and Qiemo Uygur and Xinjiang Uygur in northwest China [43,44,45]; European populations: Estonian [46], Italian [47,48,49,50,51,52], Norwegian [53,54], European American, and Hispanic American [38]; and African population: African American [38]. According to the *Rst* values, the Kyrgyz group had the closest distances to the northwest Chinese populations, Qiemo Uygur (*Rst* = 0.0831) and Xinjiang Uygur (*Rst* = 0.0840), followed by the European populations with *Rst* ranging from 0.1177 to 0.1639, and then by other populations from East Asia (the *Rst* values were in the range of 0.2199 to 0.2957). The Kyrgyz group was the farthest distant from the African Americans (*Rst* = 0.3591). The MDS and NJ tree were plotted based on *Rst* values, respectively. In the MDS plot (Figure 3b), the Kyrgyz group, located at the first quadrant, was closer to the European populations. The NJ tree (Figure 4b) showed that the African population formed the top branch by itself, the East Asian populations formed the middle branch, and the Kyrgyz, two Uygur ethnic groups, and European populations formed the bottom branch. 

### 3.6. Independence Test for Two Marker Systems

The contingency table of the two genetic marker systems containing 3828 distinct pairs of 88 individuals was listed in Table S5. Fisher’s exact tests were conducted with simulated *p*-values based on 2000 replicates. There were no statistically significant correlations between the 58 mtDNA SNPs and 24 Y-STRs (*p*-value = 0.4168), indicating that the two systems were mutually independent from each other. 

## 4. Discussion

In this study, we analyzed the genetic diversities based on the maternal inherited 60 mtDNA loci and the paternal inherited 24 Y-STRs for the Kyrgyz group in China. The haplogroup distributions for mtDNA and Y-STRs indicated the different genetic backgrounds of Kyrgyz group in maternal and paternal lineages. Also, we found that the results of the genetic relationships of the Kyrgyz group and reference populations were not completely equivalent between maternal and paternal inheritances.

Pairwise *Fst* or *Rst* values are fundamental measures to indicate the genetic distances between populations for mtDNA and Y-STRs, respectively. In our previous studies [3,4], we calculated pairwise *Fst* values among the Kyrgyz group and reference populations based on autosomal 30 InDels and 21 STRs to explore their genetic relationships in autosomal inheritance. Those results showed that Kyrgyz group had close genetic relationships to the Kazak and Uygur groups, who also lived in Xinjiang province. Then, the MDS plot for pairwise *Fst* values based on these autosomal InDels revealed that the Kyrgyz group was distributed in the middle of the plot and between East Asian and European populations [3].

In this study, we collected the genotyping data of populations worldwide as the reference populations to calculate pairwise *Fst* for mtDNA loci and pairwise *Rst* for Y-STRs. The two sets of values indicated that the Kyrgyz group had the closest genetic relationships with the ethnic groups in northwest China (the closest to the Kazak group on mtDNA markers and the closest to the Uygur group on Y chromosome markers). Beyond that, the pairwise *Fst* values for mtDNA SNPs revealed that the Kyrgyz group was genetically closer to East Asian populations, in contrast to the results from pairwise *Rst* values for Y-STRs, which showed that Kyrgyz group was genetically closer to European populations.

On the MDS plot for mtDNA (Figure 3a), the Kyrgyz and Kazak ethnic groups lied closely near East Asian populations. When it came to Y-STRs (Figure 3b), the Kyrgyz group was surrounded by European populations. We got similar results from the plots of the two NJ trees (Figure 4a,b). Compared with the paternal inheritance, the Kyrgyz group on the maternal inheritance was closer to East Asian populations, indicating some differences in their maternal and paternal evolutionary histories. Previously reported research indicated the similar pattern with the differences in maternal and paternal inheritances was also observed in the Kurdish groups by Nasidze et al. [55], which showed closer relationships with European populations than the Caucasian populations based on mtDNA but the opposite based on Y-chromosome markers.

The particular genetic structure of Kyrgyz group was related to its geographical location, historical background, and marriage customs. The Kyrgyz individuals collected by our study were from the Kizilsu Kirghiz Autonomous Prefecture in the northwest part of China, where the northern and western parts are bordered by Kyrgyzstan and Tajikistan, respectively. The area where the Kyrgyz group located was the route of the ancient Silk Road [56] and an important hub for exchanges between East Asia and Europe. This geographical location also creates an opportunity for genetic exchanges, which may be a reason to explain that the genetic structure of the Kyrgyz group is Eurasian in autosomal inheritance.

From the Han dynasty to the early Qing dynasty, the Kyrgyz people were active in the Yenisei River Basin [57]. In 1702, due to the invasion of Tsarist Russia and their fierce conflicted with the Kyrgyz people, the Junggar tribes, who ruled the area at that time, forced the Kyrgyz people to move to the Tianshan area, where they merged with a small number of Kyrgyz who had previously moved westward, and then the merged Kyrgyz people were distributed into the area surrounding Lake Issyk-Kul and the Chu and Taras river basins [58]. Subsequently, due to the oppression of the Junggar tribes, some of those Kyrgyz people moved to Tashkent, Fergana, and the surrounding areas in Central Asia, and others moved to the Pamirs, Kush Mountains, and Karakorum Mountains, forming the east–west “Brut” [59,60], which eventually evolved into the current distribution of the Kyrgyz group in China. During this long migration process, the Kyrgyz group intermarried with the surrounding ethnic groups, especially the ethnic groups from East Asia. Therefore, the ancestry components of East Asian populations were added to form the current genetic structure of the Chinese Kyrgyz group.

The Kyrgyz group adopts an outside-marriage system [61], which stipulates that individuals within seven generations of immediate family members or five generations of collateral relatives cannot be intermarried, nor can men and women who grew up drinking the same breast milk [62]. Before a couple from the Kyrgyz group gets engaged, their family and milking histories need to be known in great detail. Kyrgyz women cannot get married with men from other ethnic groups or men of different religions. However, Kyrgyz men are able to marry women from other ethnic groups, and women of different religions [63]. When a husband dies, the widow cannot take away their children and cannot change the tribe or ethnic group to which the children belong [58]. In the present study, approximate 70% of the mtDNA haplogroups in the Kyrgyz group distributed in East Asian populations based on maternal inheritance, whereas on account of Y-STR of paternal inheritance, the Kyrgyz group was closer to the European than East Asian populations. Previously reported research indicated that the Kyrgyz group had the mixture ancestry information components of East Asian and European populations in terms of autosomal genetic markers [3]. In the mitochondrial matrilineal genetic study, a very high proportion of ancestry component of the Kyrgyz group belonging to East Asian populations were mainly derived from the likely reason of intermarriage between Kyrgyz men and East Asian women according to Kyrgyz marriage custom. The Y chromosomal genetic markers strictly follow paternal inheritance, where little recombination occurs during the paternally genetic process. Thus, a large proportion of European ancestry component in Kyrgyz male individuals could still be detected in the Y-STR genetic system for paternal inheritance.

The Fisher’s exact tests indicated that the two genetic marker systems were mutually independent between mtDNA SNPs and Y-STRs. In fact, the Y chromosome DNA marker is inside the nucleus of the cell, whereas mtDNA is in the mitochondria outside the nucleus. Therefore, the two marker systems are generally considered as independent inheritances during the courses of maternal and paternal inheritances. As expected, there were no statistically significant correlations between the two marker systems in current study. Since independent inheritance is the premise for the joint application of two different marker systems, and mtDNA and Y chromosome genetic markers are always analyzed simultaneously in the field of population genetics [24,55], we presented a methodology to test for the independence between them. However, additional studies will be warranted to confirm or deny the present result. 

## 5. Conclusions

The obtained mtDNA haplogroups in this study accounted for over two thirds of the studied Kyrgyz individuals, which were also common in East Asian populations, whereas the observed Y-STR halogroups that were mainly prevalent in East Asia populations accounted for only a small fraction of a total of the obtained Y-STR haplogroups. In population genetic relationship analyses, the Kyrgyz group was genetically closer to East Asian populations than European populations based on the mtDNA marker system. However, the analyses using the Y chromosome marker system provided different results, indicating the potential differences between the maternal and paternal inheritances. In future study, more genotyping data of different genetic markers from the Kyrgyz group are needed to further reveal the genetic background of the Chinese Kyrgyz group and its genetic relationships with other populations.

## Supplementary Materials

The following are available online at https://www.mdpi.com/2073-4425/11/5/564/s1, Table S1: The allele frequencies of 60 mtDNA loci in Kyrgyz group (*n* = 138), Table S2: Haplogroup frequencies and counts for 59 mtDNA loci except (CA)n in 138 Kyrgyz individuals, Table S3: The allele frequencies and forensic parameters of 24 Y-STRs in Kyrgyz group (*n* = 241), Table S4: Haplogroup frequencies and counts for 23 Y-STRs except DYS460 in 241 Kyrgyz males, Table S5: Contingency table for independence tests between 24 Y-STRs and 58 mtDNA SNPs (*n* = 88).
